# ECT9 condensates with ECT1 and regulates plant immunity

**DOI:** 10.3389/fpls.2023.1140840

**Published:** 2023-04-11

**Authors:** Hui Wang, Ruixia Niu, Yulu Zhou, Zhijuan Tang, Guoyong Xu, Guilong Zhou

**Affiliations:** ^1^ State Key Laboratory of Hybrid Rice, Institute for Advanced Studies (IAS), Wuhan University, Wuhan, Hubei, China; ^2^ Hubei Hongshan Laboratory, Wuhan, Hubei, China

**Keywords:** RNA binding proteins, YT521-B homology domain, ECT9, ECT1, Liquid-liquid phase separation, Condensation, Plant immunity, *Arabidopsis thaliana*

## Abstract

Mounting an efficient defense against pathogens requires RNA binding proteins (RBPs) to regulate immune mRNAs transcription, splicing, export, translation, storage, and degradation. RBPs often have multiple family members, raising the question of how they coordinate to carry out diverse cellular functions. In this study, we demonstrate that EVOLUTIONARILY CONSERVED C-TERMINAL REGION 9 (ECT9), a member of the YTH protein family in Arabidopsis, can condensate with its homolog ECT1 to control immune responses. Among the 13 YTH family members screened, only ECT9 can form condensates that decrease after salicylic acid (SA) treatment. While ECT1 alone cannot form condensates, it can be recruited to ECT9 condensates *in vivo* and *in vitro*. Notably, the *ect1/9* double mutant, but not the single mutant, exhibits heightened immune responses to the avirulent pathogen. Our findings suggest that co-condensation is a mechanism by which RBP family members confer redundant functions.

## Introduction

RNA-binding proteins (RBPs) recognize RNAs through RNA-binding domains (RBDs) and modify the fates or functions of the target RNAs (Lunde et al., 2007; Hentze et al., 2018). Over 1,000 and 2,000 proteins are currently identified as RBP candidates in Arabidopsis and humans, respectively (Gerstberger et al., 2014; Marondedze et al., 2016; Marx, 2018; Zhou et al., 2021). RBPs participate in various cellular biological processes across eukaryotic cells, including growth, development, and biotic and abiotic stress responses (Glisovic et al., 2008; Lukong et al., 2008; Lorkovic, 2009; Turner and Diaz-Munoz, 2018). RBPs often have multiple family members, and it is unclear how these family members work redundantly to determine cellular activities. In this study, we focused on YT521-B homology (YTH) domain-containing proteins to address this question.

The YTH domain is a non-classical RNA-binding domain capable of recognizing RNAs modified with *N* ^6^-methyladenosine (m^6^A) (Stoilov et al., 2002; Zhang et al., 2010; Liao et al., 2018). Phylogenetic analysis revealed that the YTH domain was highly conserved in eukaryotes (Hazra et al., 2019; Shi et al., 2019; Yue et al., 2019), but little is known about plant homologs.

Thirteen YTH domain-containing proteins were identified in Arabidopsis and grouped into the YTHDF subfamily and the YTHDC subfamily (Bhat et al., 2018). Further analysis assigned eleven EVOLUTIONARILY CONSERVED C-TERMINAL REGION (ECT) homologous proteins (i.e., ECT1-ECT11) to the YTHDF subfamily and the remaining two proteins, AtCPSF30 and AT4G11970, to the YTHDC subfamily (Li et al., 2014; Yue et al., 2019). Studies of these family members in Arabidopsis mainly focused on ECT1-4 (Arribas-Hernandez et al., 2020; Wu et al., 2020; Arribas-Hernandez et al., 2021a; Arribas-Hernandez et al., 2021b). ECT1 and ECT2 interact with CBL-INTERACTING PROTEIN KINASE 1 (CIPK1) to regulate gene expression by relaying cytosolic Ca^2+^ signals to the nucleus (Ok et al., 2005). As m^6^A readers, ECT2 and ECT3, cooperating with ECT4, are required for the correct leaf formation and normal leaf morphology through an m^6^A-YTH regulatory module in the cytoplasm (Arribas-Hernandez et al., 2018; Scutenaire et al., 2018; Wei et al., 2018). These results suggest that the ECTs have functional redundant roles, leaving the question of how the redundancy occurs.

RBPs often interact with RNAs and form condensates in cells. Many RBPs with intrinsically disordered regions (IDRs) or low complexity domains (LCDs) form membrane-less organelles (MLOs) by liquid-liquid phase separation (LLPS) (Alberti et al., 2009; Li et al., 2012; Alberti, 2017; Wang et al., 2018; Bentley et al., 2019). RNA granules, including nucleoli, Cajal bodies, nuclear speckles, processing bodies, and stress granules, regulate different cellular processes spatiotemporally (Li et al., 2018; Alberti et al., 2019; Shao et al., 2022). Recent studies have shown that RBPs form biomolecular condensates by LLPS and participate in plant growth and development, signal transduction, disease resistance, and stress responses (Zhou et al., 2021; Xuan et al., 2022). The Arabidopsis YTHDC subfamily member CPSF30 recognizes m^6^A-modified far-upstream elements of flowering-relevant transcripts to control alternative polyadenylation, which occurs in liquid-like nuclear bodies (Song et al., 2021). Despite increasing studies of LLPS of YTH proteins in mammals (Gao et al., 2019; Ries et al., 2019; Fu and Zhuang, 2020; Cheng et al., 2021), little is known about plant homologs, particularly during environmental stress responses.

Here, we show that the Arabidopsis YTHDF subfamily member ECT9 (AT1G27960) undergoes LLPS *in vivo* and *in vitro*. The formation of ECT9 condensates depends on the cooperation of the IDR and YTH domains, reminiscent of human YTHDF proteins. Intriguingly, ECT1 (AT3G03950) does not form condensates solely but can be recruited to ECT9 condensates *in vivo* and *in vitro*. In addition, ECT9 works collaboratively with ECT1 to restrict the immune response to the avirulent bacterium *Pseudomonas syringae* carrying the AvirRpt2 effector, suggesting a co-condensation mechanism for RBP homologs in plants to regulate plant immunity.

## Results

### Arabidopsis ECT9 forms condensates *in vivo*

To map the domain architectures of Arabidopsis YTH family members, we performed a phylogenetic analysis of 160 YTH protein candidates from 11 different species. We consistently detected YTHDF and YTHDC subfamilies (Scutenaire et al., 2018; Yue et al., 2019). In addition, we found that plant YTH members expanded in both subfamily clades because yeasts have a single member in each subfamily (**Supplementary Figure 1A**).

**Figure 1:**
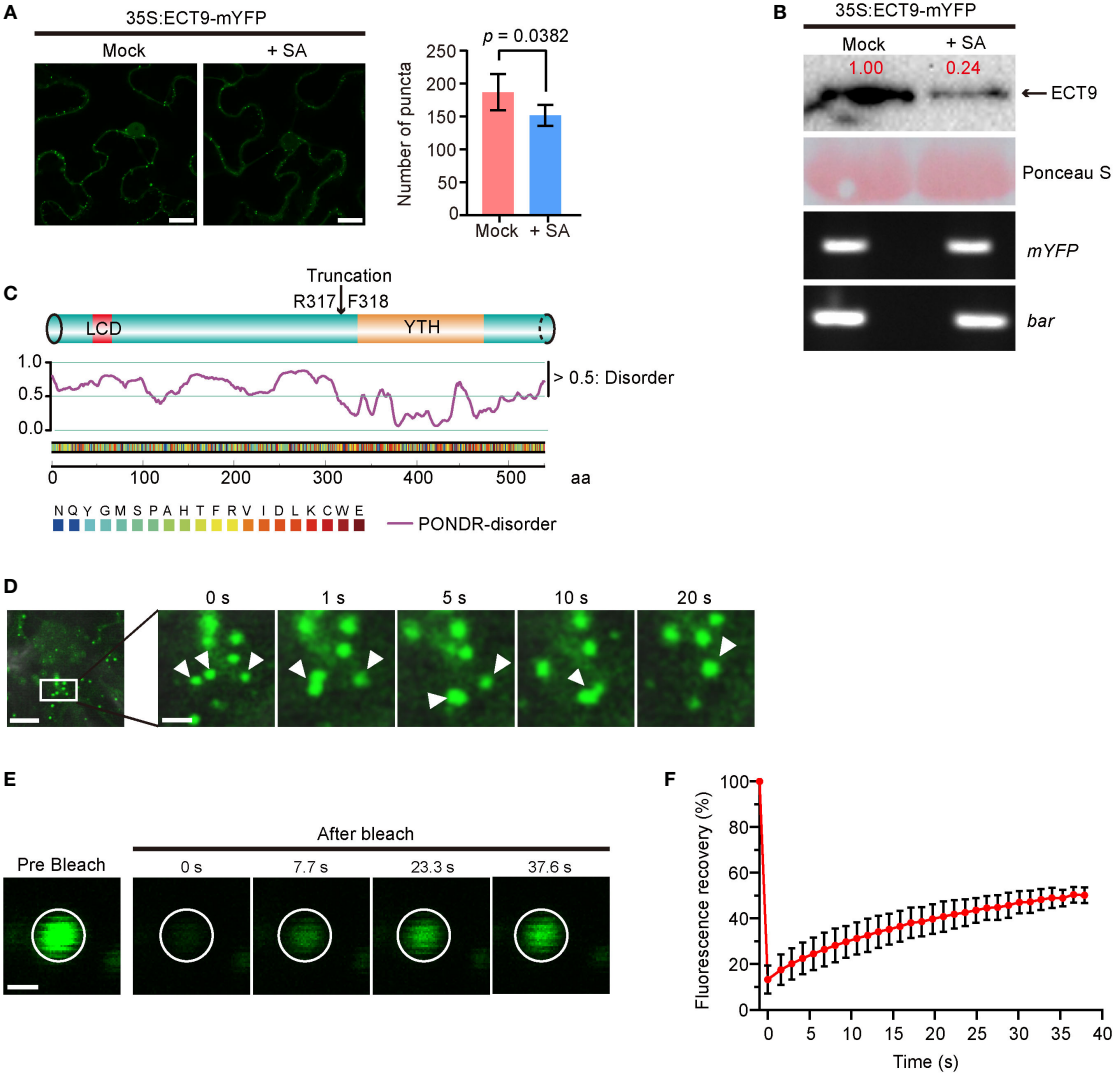
ECT9 exhibits liquid-liquid phase separation (LLPS) behavior *in vivo* and responds to salicylic acid (SA). **(A)** Number of puncta in *N. benthamiana* leaves expressing *35S:ECT9-mYFP* after 3-hour treatment with 2 mM SA. Mean ± SD (n = 5). Mock, H_2_O. Scale bar, 10 µm. *p* value by Two-sided Student’s *t*-test. **(B)** Protein and mRNA expression levels of *ECT9* in **(A)**. Relative value of protein was marked by Image Lab. Ponceau S, protein loading control. *bar*, the selection marker gene *basta* on the *35S:ECT9-mYFP* binary T-DNA vector. **(C)** Disordered region prediction of ECT9. Top, schematic diagram of protein domains of ECT9 from UniProt. Bottom, disordered score predicted by ‘Predictor of Natural Disordered Regions’ (PONDR; http://www.pondr.com/). Scores above 0.5 indicate disordered regions. **(D)** Fluorescence time-lapse microscopy of *N. benthamiana* leaves expressing *35S:ECT9-mYFP*. Three fusing bodies are zoomed in. Scale bar, 10 µm (left) and 2.5 µm (right). **(E)** FRAP of ECT9-mYFP condensates. Time 0 indicates the start of the photobleaching pulse. Scale bar, 2 µm. **(F)** A plot showing the time course of the fluorescence recovery after photobleaching mYFP-ECT9 condensate. Mean ± SD (n = 8). Data are representative of three independent experiments.

To reveal how Arabidopsis YTH proteins behave redundantly, we first examined their subcellular localization. We transiently expressed Arabidopsis YTH proteins in *N. benthamiana* leaves and tested their responsiveness to the immune signal SA. We found that three proteins (i.e., ECT4, ECT8 and CPSF30) were specifically localized in the nucleus, and the remaining proteins were distributed in the cytosol and nucleus (**Supplementary Figures 1B, C**). They displayed even distribution except for ECT9, which formed puncta before SA treatment (**Figure 1A**). We then performed an observation of ECT9 with different subcellular markers. We used UBP1b to label the bodies in the nucleus and cytosol, G3BP1 to label the bodies in the cytosol, and FCA to label the bodies in the nucleus (Nguyen et al., 2016; Fang et al., 2019; Abulfaraj et al., 2021; Reuper et al., 2021). We found that ECT9 puncta co-localized with all these body markers, indicating that ECT9 is a component of these bodies (**Supplementary Figure 2A**).

We observed that the number of ECT9 puncta was significantly reduced when the *N. benthamiana* leaves expressing *ECT9-mYFP* were treated with 2 mM SA. However, we did not detect noticeable changes in the YFP signals of the other YTH family members after SA treatment (**Supplementary Figure 1B**). To find out how ECT9 puncta disappeared, we measured the protein level of ECT9 and detected a corresponding reduction (**Figure 1B**). This reduction at the protein level was not due to the SA effect on ECT9 transcription because we did not detect any mRNA changes during SA treatment (**Figure 1B**). We further confirmed the sensitivity of ECT9 to SA in Arabidopsis mesophyll protoplasts, and a similar result was observed after SA treatment (**Supplementary Figure 2B**).

Since RBPs containing a PrLD (Prion-Like Domain) or LCD were prone to form granules or fuse into droplets in cells through LLPS, we then predicted the disordered structure of ECT9 protein by the PONDR website (“http://www.pondr.com/“). A disordered region was found at the N-terminal of ECT9 (**Figure 1C**), indicating that ECT9 has a potential for LLPS. Surprisingly, other members also have predicted disordered regions, suggesting that carrying the predicted disordered region is insufficient to form condensates *in vivo* (**Supplementary Figures 1B, C**). It is also possible that these family members form condensates in other unknown conditions.

To test whether the ECT9 puncta formed in *N. benthamiana* leaves resulted from LLPS, we explored the puncta’s fluidity, reversibility, and fusion properties. Using time-lapse microscopy, we found that ECT9 puncta could fuse (**Figure 1D**). We then assessed the dynamic of ECT9 puncta by the fluorescence recovery after the photobleaching (FRAP) assay. The spatiotemporal analysis of bleaching events showed that ECT9 redistributed rapidly from the unbleached area to the bleached area (**Figures 1E, F**). We conclude that ECT9 localizes to the nuclear and cytoplasmic bodies with liquid-like properties, suggesting that ECT9 can undergo LLPS *in vivo*.

### Full-length ECT9 undergoes phase separation *in vitro*

**Figure 2:**
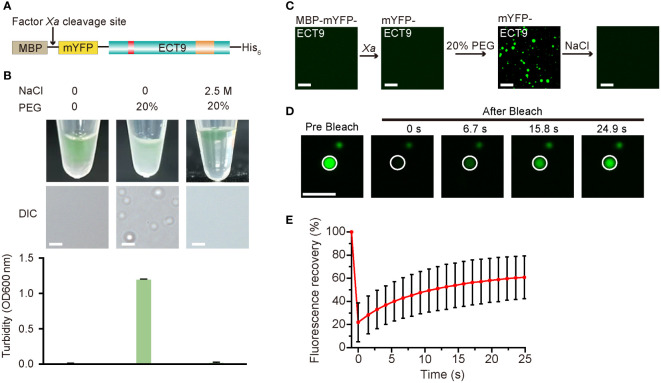
Full-length ECT9 undergoes phase separation *in vitro*. **(A)** Schematic of the full-length protein fusion used for *in vitro* phase separation assay. **(B)** Phase separation behaviors of purified mYFP-ECT9 (28 µM) in the presence of crowding agent PEG with or without 2.5 M NaCl by a white light photograph in tubes (top) and DIC microscopy observation for droplet formation (middle) and optical density measurement at 600 nm (OD_600_ nm) of turbidity changes (bottom). Turbidity data are presented as means ± SD of OD_600 nm_ value (n = 4). Scale bar, 50 µm. **(C)** *In vitro* phase separation assay of mYFP-ECT9 by a confocal laser scanning microscopy photograph. Scale bar, 20 µm. **(D)** FRAP of mYFP-ECT9 condensates *in vitro*. Time 0 indicates the start of the photobleaching pulse. Scale bar, 1 µm. **(E)** A plot showing the time course of the fluorescence recovery after photobleaching mYFP-ECT9 droplets. Mean ± SD (n = 8). Data are representative of three independent experiments.

We investigated whether ECT9 is capable of undergoing LLPS *in vitro*. Specifically, we expressed the recombinant full-length ECT9 fusion protein (MBP-mYFP-ECT9) in *E. coli Rossetta* (DE3) and purified it to a high homogeneity (**Figure 2A**). After digesting the purified protein with Factor *Xa* to remove the MBP tag, we used the purified mYFP-ECT9 fusion protein to examine phase separation behavior *in vitro*. We observed pronounced phase separation of mYFP-ECT9 at 28 µM, which was illustrated by enhanced turbidity and the formation of spherical droplets in the presence of the crowding agent that strengthened intermolecular interaction (**Figure 2B**). However, mYFP-ECT9 did not undergo phase separation at a high salt concentration that disrupted such interaction (**Figure 2B**). Confocal microscopy allowed us to observe the high-resolution results (**Figure 2C**). We also performed FRAP experiments to observe the mobility of ECT9 within the droplets. FRAP analysis indicated that mYFP-ECT9 molecules diffused rapidly within droplets and exchanged between droplets and the surrounding solution. The curve plot showed that the intensity of the mYFP labeled ECT9 fluorescence signal recovered over 50% within 30 s after photobleaching (**Figures 2D, E**). Together, these data indicate that mYFP-ECT9 undergoes LLPS *in vitro*.

To determine the functional structure required for ECT9 droplet formation *in vivo* and *in vitro*, we conducted a truncation screen for ECT9 function. We expressed the N-terminal disordered domain (ECT9N, 1-317 aa) or C-terminal YTH domain (ECT9C, 318-539 aa) separately in the *E. coli* DE3 strain (**Supplementary Figure 3A**). After cleaving the MBP tag with Factor *Xa*, we found that neither mYFP-ECT9N nor mYFP-ECT9C formed spherical droplets. Instead, we only observed fiber-like aggregates under the confocal microscope with the addition of 20% PEG8000 (**Supplementary Figure 3B**). These aggregates were fibrotic and non-liquid since the FRAP assay did not capture fluorescence recovery after bleaching them (**Supplementary Figure 3C**). The result suggests that the ability of phase separation to form liquid-like droplets *in vitro* depends on the intact structure of the ECT9 protein. We also transiently expressed ECT9 truncation in *N. benthamiana* leaves and found that ECT9N and ECT9C proteins had different subcellular localizations compared to the full-length ECT9 protein. Specifically, ECT9N was detected in the cytosol and nucleus, while ECT9C was mainly observed in the nucleus (**Supplementary Figure 3D**). Furthermore, we did not observe puncta formation of ECT9N or ECT9C (**Supplementary Figure 3D**). These results indicate that both N-terminal and C-terminal structures are required for the condensation behavior of ECT9 *in vivo*.

### ECT9 interacts and co-condensates with ECT1

**Figure 3:**
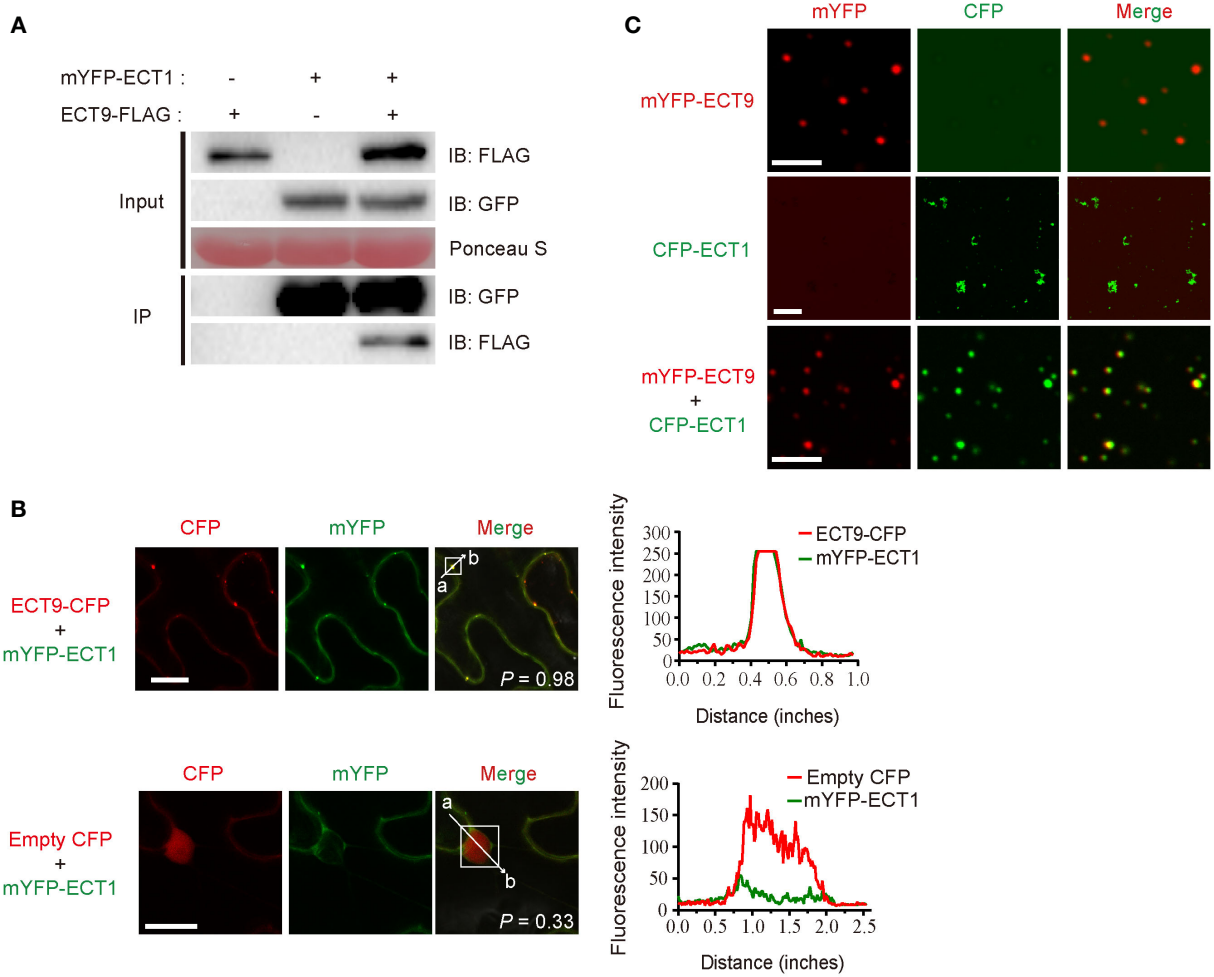
ECT9 directly interacts and co-phase separates with ECT1. **(A)** Co-IP assay showing the interaction between ECT9 and ECT1 expressed in *N. benthamiana* leaves. Extracted proteins were incubated with GFP-trap agarose beads. The immunoprecipitates were analyzed by immunoblotting (IB) with anti-GFP or anti-FLAG antibodies. **(B)** Co-localization of ECT9 with ECT1.The mYFP-ECT1 fusion proteins were co-expressed with ECT9-CFP or empty CFP fusions in *N. benthamiana* leaves. Scale bar, 20 µm. The right panels show plots of relative CFP (red) or mYFP (green) fluorescence intensity along the line from a to b depicted in the corresponding merged images. Solid white line rectangles indicate the area used to calculate the co-localization score (*P*, Pearson’s *R*-value) depicted at the bottom-left of merged images. **(C)** Co-condensation of ECT9 with ECT1. The purified proteins were isolated or mixed for *in vitro* phase separation (induced condition: 32 µM mYFP-ECT9 and/or CFP-ECT1, 20% PEG8000). Scale bar, 8 µm (top and bottom) and 25 µm (middle). Data are representative of three independent experiments.

To investigate how ECT9 interplays with other family members, we examined if ECT9 interacted with other homologs to form a protein complex that enables the formation of heterotypic condensates. We screened the interaction between ECT9 and the other 12 members by performing the Split Luciferase Complementation Assay (SLCA). The fluorescence signal was mainly detected by combining nLUC-ECT9 with cLUC-ECT1 but not others (**Supplementary Table 4**). We then confirmed the interaction between ECT9 and ECT1 *in vivo*. We performed the co-immunoprecipitation (Co-IP) assay in planta. Proteins were extracted from *N. benthamiana* leaves expressing *ECT9-FLAG* and *mYFP-ECT1* constructs. We used the GFP-trap beads to capture the interactor of ECT1 and detected ECT9 in the IP complex, suggesting that ECT9 interacted with ECT1 in planta (**Figure 3A**). We then performed colocalization between ECT9 and ECT1 in cells. We showed that ECT1 was diffused in the cytosol (**Supplementary Figure 1C**). However, in the co-localization assay, we found a completely overlapping fluorescence signal of ECT9 and ECT1 in the cytosol in a condensate form (**Figure 3B**). It suggests that ECT9 interacts with ECT1 and recruits it into the co-existed condensates.

To validate the relevancy of co-condensation of ECT9 and ECT1, we expressed and purified recombinant fusions of ECT9 and ECT1 and then performed the phase separation assay *in vitro*. We found that under the same protein and PEG concentrations, ECT1 formed fiber-like aggregates similar to ECT9N and ECT9C, different from the typical spherical liquid-like droplets of the full-length ECT9 (**Figure 3C**). Surprisingly, we observed the spherical liquid-like droplets of ECT1 when we mixed it with ECT9 (**Figure 3C**). It was not due to the protein or PEG concentration since we controlled their final concentration to be the same as separate tests. The result was in line with our observation of the co-localization of ECT9 and ECT1 in the puncta in planta. Together, these results suggest that ECT9 affords condensation capability to the incapable ECT1.

### ECT9 and ECT1 play a negative role in plant immunity

To test whether the cooperation of these two family members also acted during immune responses, we generated *ECT1* and *ECT9* knockout lines in Col-0 background using the CRISPR system (*ect1* and *ect9*; **Supplementary Figure 4A**). We backcrossed these lines with Col-0 three times to remove the potential off-target effects. We deleted 922 bp from -52 bp before the ATG start codon of *ECT1*, and the residual *ect1* mRNA was unlikely to produce functional proteins (**Supplementary Figures 4A, B**). We also deleted 1791 bp within the genomic DNA of *ECT9*, and the residual *ect9* mRNA was very low (**Supplementary Figures 4A, B**). We then crossed *ect1* into *ect9* and obtained the double mutant *ect1/9*. Expression analysis showed that the expression of *ECT9* and *ECT1* was dramatically decreased in the double mutant (**Supplementary Figure 4B**). We did not notice any growth or developmental defects in the single or double mutants compared to the wild type (WT; **Supplementary Figure 4C**). It suggests that ECT1/9 are not involved in plant growth and development or that other ECT family members are increased to support the ECT1/9 deficiency in the double mutant.

We then investigated the defense responses of *ect1* and *ect9* plants to the virulent bacterial pathogen *Psm* ES4326. The *ect1* mutant did not differ from the WT plants, while the *ect9* mutant exhibited slight resistance to *Psm* ES4326 despite no statistical significance (**Supplementary Figure 5A**). We found that the *ect1/9* double mutant showed higher resistance to *Psm* ES4326 (**Supplementary Figure 5A**). However, the variation among individuals caused no statistical significance. It suggests that *ECT1* and *ECT9* are not the essential immune components in basal resistance.

To elucidate the role of *ECT1* and *ECT9* in plant innate immunity, we tested the defense responses in pattern-triggered immunity (PTI) and effector-triggered immunity (ETI). We activated PTI responses by infiltrating plants with bacterial epitope elf18, a microbe-associated molecular pattern that can be recognized by the pattern-recognition receptor EFR. We observed a complete PTI response in the single or double mutants by examining bacterial growth and callose deposition (**Supplementary Figures 5B, C**). Therefore, *ECT1* and *ECT9* are not essential immune components in PTI response.

**Figure 4:**
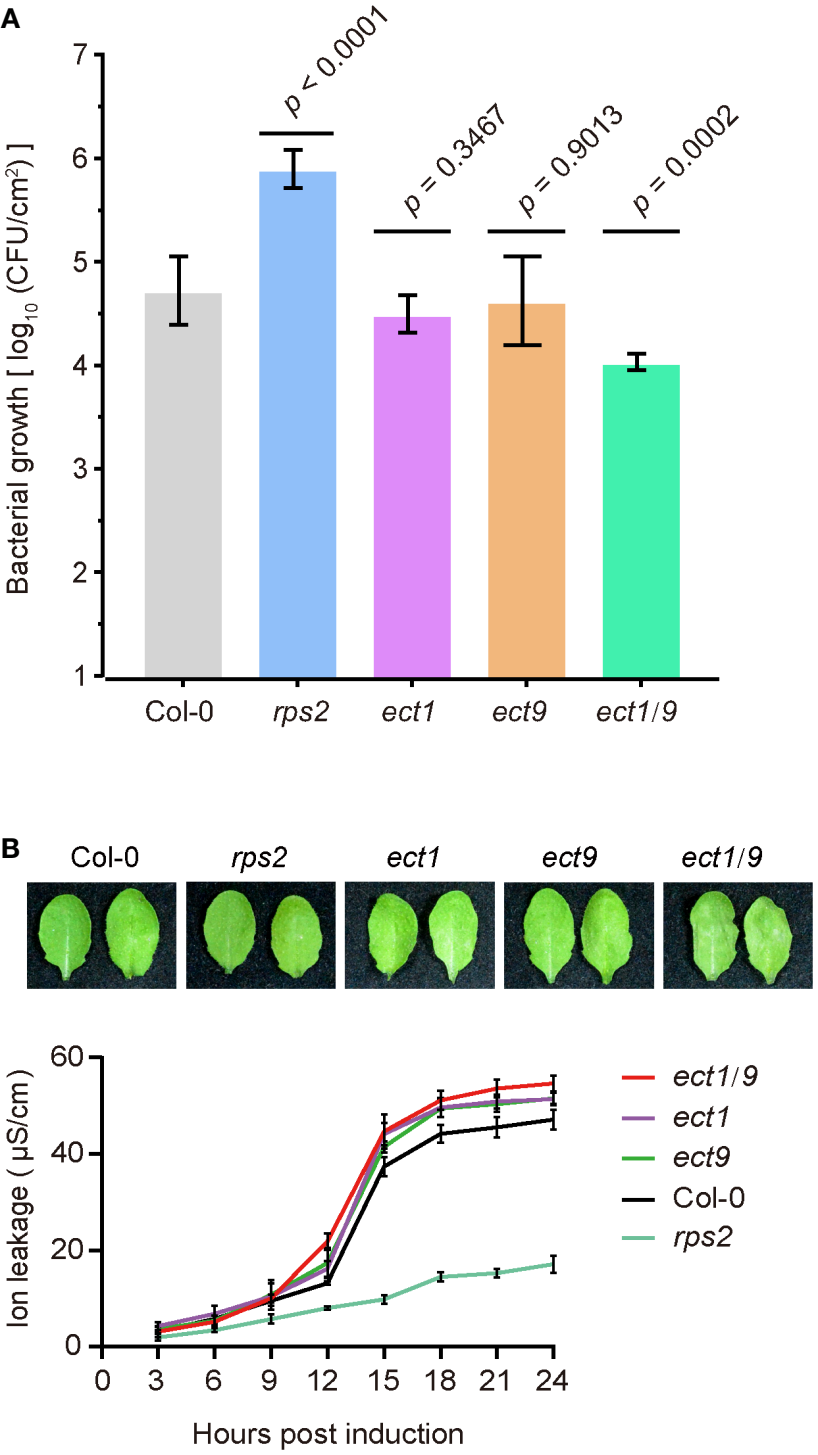
ECT9 and ECT1 play a negative role in resistance to *Psm* ES4326 (AvrRpt2). **(A)** *Psm* ES4326 (AvrRpt2) was infiltrated into Arabidopsis leaves at OD_600 nm_ = 0.001. Bacterial growth was determined at 3 dpi (mean ± SD; n = 7). Data were analyzed using one-way ANOVA with Dunnett’s test. dpi, days post-inoculation. **(B)** Cell death in Col-0, *ect1*, *ect9*, *ect1/9*, and *rps2* mutants by photographs (top) and the conductivity assay (bottom). Conductivity measurements were performed every 3 hours after infiltration with *Psm* ES4326 (AvrRpt2). Mean ± SD (n = 4). Data are representative of three independent experiments.

We then tested the ETI responses by testing bacterial growth after infiltrating the avirulent pathogen *Psm* ES4326 (AvrRpt2). We found that the *rps2* mutant had a dramatic bacterial growth because of the defect in recognizing the AvrRpt2 effector. However, we found that the *ect1* or *ect9* single mutant did not differ in bacterial growth compared to the WT (**Figure 4A**). In contrast, the *ect1/9* double mutant exhibited reduced bacterial populations, showing a resistance to the pathogen (**Figure 4A**). We then transformed the native promoter-driven ECT9-mYFP (*Pro_ECT9_:ECT9-mYFP*) into the *ect1/9* double mutant and tested the bacterial growth on multiple T1 lines. The complementation lines have similar bacterial growth to the *ect1* single mutant, suggesting that the fusion protein of ECT9-mYFP is functional (**Supplementary Figure 5D**). Increased ETI resistance is usually accompanied by enhanced hypersensitive response cell death. We examined the cell death rate by measuring the ion leakage in these mutants. We detected a minor increase in electrolyte leakage in the *ect1/9* double mutant (**Figure 4B**). These results suggest that *ECT9* and *ECT1* play a negative role in ETI response in a redundant manner.

### Transcriptome analysis of *ect1/9* during ETI activation

**Figure 5:**
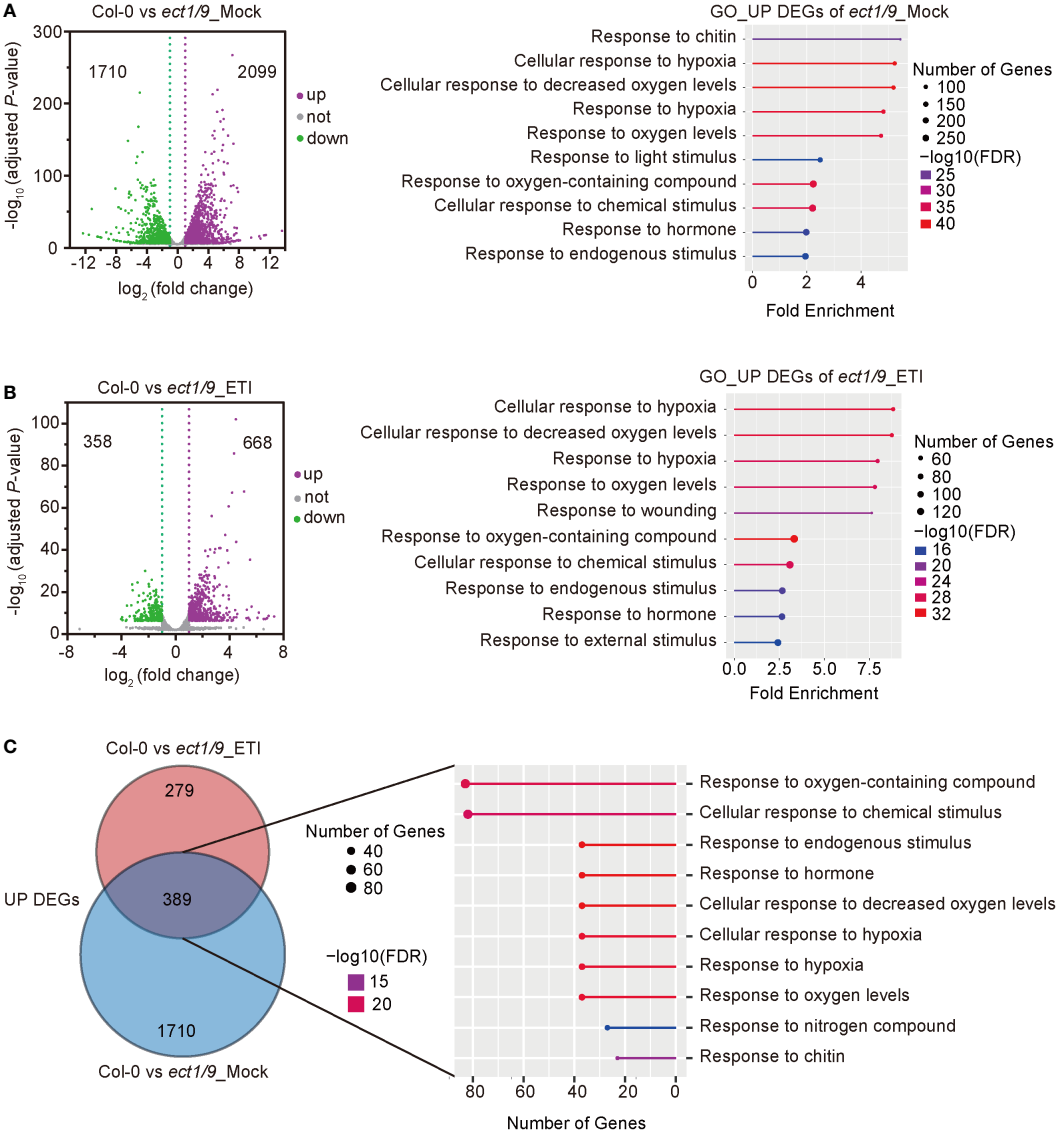
RNA-seq analysis of *ect1/9*. **(A)** Analysis of *ect1/9* without ETI activation (Mock). The volcano plot shows differentially expressed genes (DEGs) based on two replicates of RNA-seq data in the *ect1/9* double mutant compared with the wild type Col-0. Violet dots represent up-regulated DEGs, green dots represent down-regulated DEGs, and gray dots represent the genes with no significant change. The y-axis shows -log_10_ (adjusted *p*-value), while the x-axis shows log_2_(fold change) values. Right, the top 10 significantly enriched Gene Ontology (GO) terms of up-regulated DEGs in the *ect1/9* double mutant (FDR < 0.05). The color of the square indicates the significance of the term. The size of each point corresponds to the gene number of each GO term. **(B)** Analysis of *ect1/9* with ETI activation by *Psm* ES4326 (AvrRpt2). **(C)** Venn diagrams show the overlaps between up-regulated DEGs in the *ect1/9* double mutant with (ETI) and without (Mock) *Psm* ES4326 (AvrRpt2) treatment. The numbers of overlapping DEGs were further used for GO analysis. The top 10 significantly enriched GO terms shared of up-regulated DEGs were shown in the right panel.

To explore the ECT1/9-mediated regulatory network in response to *Psm* ES4326 (AvrRpt2), we performed an RNA-seq analysis using the 4-week-old WT and *ect1/9* double mutant with the treatment of Mock (H_2_O) and *Psm* ES4326 (AvrRpt2). We calculated the Pearson correlation coefficient (*r*) for each pair of the two biological replicates for genes with the fragments per kilobase million (FPKM) of protein-coding genes (FPKM > 1). The high correlation coefficients of the two biological replicates (*r* = 0.98) indicated a reproducibility (**Supplementary Figure 6**). Compared with the WT, 3809 differentially expressed genes (DEGs), including 2099 up-regulated and 1710 down-regulated genes, were detected in *ect1/9* double mutant with the Mock treatment. Gene ontology (GO) analysis showed that defense-related genes and hypoxia-related genes were significantly enriched in the up-regulated genes (FDR < 0.05; **Figure 5A**). These results may account for a slight basal defense against the virulent *Psm* ES4326 observed in the *ect1/9* double mutant. Furthermore, 668 up-regulated and 358 down-regulated genes were detected in *ect1/9* plants versus WT plants after *Psm* ES4326 (AvrRpt2) inoculation. GO categories revealed that up-regulated genes were relevant to defense responses (**Figure 5B**). Many defense genes such as *NPR3*, *RBP-DR1*, *PEPR1*, *LHY*, *DAR5* and hypoxia-related genes *LOX1*, *LOX3*, *RD20*, *SQP1*, *MO1*, significantly increased their expression under ETI treatment (**Supplementary Table 3**). Further analysis showed that an overlapping set of 389 genes were observed in the up-regulated genes of the *ect1/9* plants with or without ETI treatment, which also enriched genes in the defense response pathways (**Figure 5C**). These data suggest that *ECT1* and *ECT9* restrict immune gene expression.

## Discussion

RBPs are often referred to as “RNA clothes” as they regulate various aspects of RNA function from production to degradation (Marx, 2018). In this regard, there are usually multiple homologs within a family to secure the redundant role of RBPs. This study aimed to answer the question of how RBP family members achieve these redundant biological functions, and ends with the discovery of ECT9 and ECT1 co-condensation as a potential mechanism.

### Phase separation behavior of YTH family proteins

Previous studies have shown that mammal YTHDF and YTHDC family members can undergo phase separation (Ries et al., 2019; Wang et al., 2020; Cheng et al., 2021). However, this characteristic is rarely studied in plant YTH proteins. Recently, an Arabidopsis YTHDC family member, CPSF30-L (also known as a polyadenylation factor), was characterized as an m^6^A reader, and the m^6^A-binding activity of CPSF30-L enhances the formation of liquid-like nuclear bodies where the mRNA polyadenylation occurs (Song et al., 2021). This study shows that an Arabidopsis YTHDF subfamily member ECT9 can undergo phase separation *in vivo* and *in vitro* (**Figures 1**, **2**). It has been reported that IDRs of RBPs are one of the main driving forces for phase separation. In our study, the disordered region of ECT9 (ECT9N) alone could not form liquid-like droplets *in vivo* and *in vitro*, and the YTH domain of ECT9 (ECT9C) alone was also unable to phase separate. The phase separation of ECT9 requires the participation of the full-length amino acid sequence, similar to previous research on human disease-related protein FUS, whose LLPS requires both the N-terminal PrLD and the C-terminal RBD (Wang et al., 2018). ECT1 cannot undergo LLPS, but it can co-condensate with ECT9. This co-condensation was also observed for Arabidopsis ATG3, which facilitates the LLPS of ATG8e to promote autophagy (Guan and Xue, 2022). Hence, ECT9 might serve as a helper to accelerate the phase transition of other ECTs proteins to respond to environmental stresses.

We analyzed the liquid-liquid phase separation ability of ECT9 in its native condition using the transgenic line transformed with the native promoter-driven ECT9-mYFP (Pro_ECT9_:ECT9-mYFP/*ect9*). Unfortunately, we did not observe this phase separation in leaves, roots, or mesophyll cells, with or without SA treatment. We also tried several independent transgenic lines with similar negative results. We attributed the following reasons. First, *ECT9* has a specific expression pattern depending on different tissues and growth and development stages. Second, ECT9 phase separation in the native condition requires a specific signal, a phase separation partner or varying stimulus strength. Third, ECT9 has multiple homologs, causing a low expression level in the complementation lines. Accordingly, we did not observe any immune phenotype on the *ect9* single mutant (**Figure 4A**, **Supplementary Figure 6A**). Fourth, ECT9 condensation may require a specific genetic background to favor its protein accumulation. In the literature, researchers have discovered that the nuclear bodies formed by the AtCPSF30 under its native promoter were unsuitable for photobleaching due to a limited protein level (Song et al., 2021). Nevertheless, we thought that the ECT9 condensates exist, because, under the same expression conditions, only ECT9, instead of the other 12 proteins, showed LLPS condensates *in vivo* (**Supplementary Figure 1C**). Also, we found that ECT9 but not ECT1 formed LLPS condensates *in vitro* under the same conditions of protein and PEG concentration (**Figure 3C**). More work is required to find the situations under which ECT9 condensates will be observed under the native condition.

### ECT9 and ECT1 play a synergistic inhibitory role in the immune response

It is hypothesized that the full-length ECT9 protein recognizes and binds the m^6^A-modified RNA *via* its YTH domain. Then it folds into the correct conformation with the N-terminal disordered region. ECT9 perhaps interacts with other factors by forming liquid-like droplets to promote the degradation of these co-factors. Accordingly, after SA treatment, fewer ECT9 condensates were observed (**Figure 1**). This mechanism is suggested to be responsible for the function of ECT9 and ECT1 in plant immunity. In WT plants, nucleic and cytoplasmic bodies formed by ECT9 could absorb immune mRNAs to avoid overactivation. However, in the *ect1/9* double mutant, these immune mRNAs are likely less engaged by the ECT9-associated bodies. These immune mRNAs continue to be transcribed and translated, resulting in elevated levels of immune proteins that increase resistance to the phytopathogen. Accordingly, the RNA-seq data analysis shows the up-regulated genes in the *ect1/9* mutant are involved in defense responses (**Figures 4**, **5**). Recent studies show that translational reprogramming is a fundamental layer of immune regulation. It remains possible that the elevated immune mRNAs undergo selective translation (Xu et al., 2017; Chen et al., 2023; Zhou et al., 2023).

It is possible that some mRNA regulatory elements, such as linear motifs, unique structures, or nucleotide modifications, mark these immune mRNAs for recognition by the ECT9-associated bodies. The nucleotide modification with m^6^A is a potential selective regulatory element since mammalian ECT9 homologs recognize mRNAs with m^6^A modification. Further investigation is required to determine whether these immune mRNAs up-regulated in the ect1/9 mutant have hallmarks of m^6^A modification. We preliminarily searched these immune mRNAs in the plant m^6^A database (http://180.208.58.19/m6A-Atlas/#) and found some methylated genes. Therefore, more efforts are required to investigate the selectivity of mRNAs by ECT9, such as using MeRIP-seq or DART-seq (Dominissini et al., 2012; Meyer, 2019).

Our study has some uncertain flaws. We did not show a direct connection between ECT9 condensation and defense responses. It would be best to complete the *ect1/9* mutant with a condensation-incapable form of ECT9. It is assumed that the enhanced resistance could not be reversed but could be achieved by the full-length ECT9. Although ECT9N and ECT9C lose the typical LLPS behaviors that the full-length ECT9 has, the complementation of the *ect1/9* mutant with these two truncation versions cannot faithfully demonstrate the direct contribution of ECT9 condensation to immune responses due to the large deletion in these two truncation forms. We expected to find some key point mutations that compromise ECT9 condensation but minimize their effect on the whole structure.

If ECT9-mediated co-condensation of ECT1 is involved in the immune responses, it is unlikely that ECT9 is the sole or dominant condensation protein. If ECT9 is the dominant one, we would have observed the elevated resistance in the *ect9* single mutant. Therefore, other RBPs must be able to mediate the condensation of ECT1 in the absence of ECT9. Accordingly, we found that ECT9 was co-localized with UBP1b, a nuclear and cytosolic body maker. We previously demonstrated UBP1b homolog UBP1c as a condensation-capable RBP using *in vivo* and *in vitro* assays (Zhou et al., 2021). This assumption aligns with the general recognition that RBPs must work redundantly to secure the mRNA fate, the essential process of delivering genetic information.

## Materials and methods

### Plant materials and growth conditions

All *A. thaliana* plants used in this study are in the Columbia-0 (Col-0) background. The *ect1* and *ect9* mutants were constructed using the CRISPR-Cas9 editing system (see section: The CRISPR-Cas9 editing system). The double mutant *ect1/9* was generated by the genetic cross and verified by PCR. The seeds were surface-sterilized and soaked in 1% Plant Preservative Mixture (PPM) and kept at 4°C in the dark for 2-3 days for vernalization, following sown on 1/2 Murashige and Skoog (MS) medium (0.22% MS, 0.05% MES, 1% sucrose, 0.8% agar, pH 5.8) plates and cultivated in the greenhouse for 6-8 days at 22 °C before being transferred to soil grown under long photoperiod conditions (16-h light of 130 µmol•m-2•s-1/8-h dark). The bacterial growth assay used a short-day (SD) condition (12-h light/12-h dark). *N. benthamiana* plants used for transient expression were grown on soil (Pindstrup, Denmark) in a phytotron at 22 °C under a 12-h light/12-h dark photoperiod with 55% relative humidity.

### Plasmid construction and genetic transformation

This study used a zero-background ligation-independent cloning procedure for vector construction, as described previously (Zhou et al., 2021). Two primary vector conformations (*X-mYFP* and *mYFP-X*) were used for construction of *N. benthamiana* transient transformation vectors. The PCR products of 13 YTH domain-containing genes coding sequences (*ECT1*-*ECT11*, *CPSF30*, *AT4G11970*) were amplified from Col-0 cDNA and alternatively cloned into primary vectors with different adaptors. For the co-localization and Co-IP assay, the full-length CDS of *ECT9* and other marker genes, *UBP1b* (AT1G17370), *G3BP1* (AT5G48650) and *FCA* (AT4G16280), were amplified and cloned into the pZT219-FLAG, pZT158-CFP and pZT156-mYFP vectors to generate the *35S:ECT9-FLAG*, *35S:ECT9-CFP*, *35S:UBP1b-mYFP*, *35S:G3BP1-mYFP* and *35S:FCA-mYFP* constructs. For SLCA assay, the CDS of *ECT9* was cloned into nLUC-pMR78 and other YTHs’ CDS were cloned into cLUC-pMR31 or pGX12-cLUC. To construct truncated *ECT9* vectors, a truncated N-terminal fragment (*ECT9N*, 1-317 aa) and C-terminal fragment (*ECT9C*, 318-539 aa) were amplified and cloned into the pJG054 and pYL181 vectors for subcellular localization and phase separation experiments, respectively. For constructing *ECT1* and *ECT9* mutants, the CRISPR-Cas9 editing system was used. Two single-guide RNA of *ECT1* and *ECT9* were designed and further cloned into the pHEE401E vector by the BsaI-Golden Gate Assembly strategy (NEB, E1601L). The *A. thaliana* transgenic plants were generated by the *Agrobacterium*-mediated floral dipping method (Zhang et al., 2006). All plasmids and primers mentioned above are listed in **Supplemental Table 1**.

### The CRISPR-Cas9 editing system

To construct CRISPR knockout mutants, we designed two guide RNA (gRNA) sequences for each gene using CRISPR-P (“http://crispr.hzau.edu.cn/CRISPR2/“) and expressed them under Arabidopsis *U6-26* (AT3G13855) and *U6-29* (AT5G46315) promoter, respectively (Liu et al., 2017). The vector pHEE401E was used as an acceptor vector. The CRISPR-associated endonuclease zCas9 was from the *Zea mays*, driven by Arabidopsis *EC1.1* (AT1G76750) promoter (Xing et al., 2014). Homozygous transgenic plants were identified using PCR-based and sequenced genotyping. All primers used for genotyping are listed in **Supplemental Table 1**.

### Phylogenetic analysis

Amino acid sequences of 160 YTH domain-containing proteins from 11 species were collected, including six monocotyledons (*Oryza sativa*, *Zea mays*, *Triticum aestivum*, *Hordeum vulgare*, *Brachypodium distachyon*, *Sorghum bicolor*), three dicotyledons (*Arabidopsis thaliana*, *Glycine max*, *Gossypium raimondii*), mammal (*Homo sapiens*), and Yeast (*Saccharomyces cerevisiae* and *Schizosaccharomyces pombe*). YTH domain sequences were aligned by the ClustalW method with the default settings in MEGA, and the phylogenetic tree was constructed by the Neighbor-Joining model with a default setting using a post-aligned document. For phylogenetic analysis among 13 Arabidopsis YTH domain-containing proteins, full-length protein sequences were used to align by the ClustalW method. The resultant Neighbor-Joining evolutionary tree was beautified on Evolview version 2 (“http://www.evolgenius.info/evolview/#/“) (Subramanian et al., 2019). The protein domain sequences and DNA sequences were organized from UniProt and TAIR websites, respectively. The disordered score was predicted from the PONDR website (“http://www.pondr.com/“) with a VSL2 predictor. The full-length amino acid sequences and YTH domain sequences used for alignment are listed in **Supplemental Table 2**.

### Subcellular localization and confocal observation

To determine the subcellular localization of Arabidopsis YTH domain-containing proteins, 4-week-old *N. benthamiana* leaves were infiltrated with *Agrobacterium tumefaciens* GV3101 strain carrying 13 transient transformation vectors. Fluorescence images were photographed 48 hours post-infiltration (hpi) using LEICA TCS SP8 laser scanning confocal microscope (Leica). For co-localization of ECT9 with ECT1 and body marker proteins, *35S:ECT9-CFP* and corresponding vectors transformed separately into *Agrobacterium* GV3101 by liquid nitrogen freezing and thawing method and co-expressed in *N. benthamiana* leaves and further imaged. At least three leaves per construct on 2-3 plants in each experiment and at least 3 repeats on different days were conducted for confocal imaging.

Arabidopsis mesophyll protoplasts were prepared as described previously and transformed with ECT9 fused mYFP fluorescent protein (Yoo et al., 2007). After incubation for 18 h in darkness at 22 °C, a final concentration of 2 mM SA or H_2_O (Mock) was added into 1 mL protoplast culture for another 3 h incubation at the same conditions and photos were taken by confocal microscope (Leica). We used the following settings for excitation/emission of fluorescence: YFP (514/525-580 nm) and CFP (448/458-505 nm). Images were analyzed using Leica Application Suite X software 3.5.2.18963 and Fiji (ImageJ v2.3.0).

### Co−immunoprecipitation and immunoblotting

The combinations of ECT9-FLAG and mYFP-ECT1 were co-transfected into *N. benthamiana* leaves. After 48 hours, the total proteins were extracted with lysis buffer (50 mM Tris-HCl, pH 7.4, 150 mM NaCl, 0.2% NP40, 0.1% Triton-100, 20 mM DTT and 1 mM PMSF) supplemented with 1 × complete protease inhibitor cocktail (Roche). After centrifugation, the supernatant was incubated with 20 µL GFP-Trap^®^ _A beads (Chromotek) for 2 h at 4°C. Then the purified proteins were detected using immunoblotting with anti-GFP antibody (ProteinGene, 2057) and anti-FLAG antibody (ProteinGene, 2064), respectively. Chemiluminescence signals were detected using the ChemiDoc ™ XRS+ imaging system (BIO-RAD).

To evaluate the effect of SA on ECT9 protein levels *in vitro*, *N. benthamiana* leaves infiltrated with *Agrobacterium* GV3101 carrying *35S:ECT9-mYFP* were sprayed with 2 mM SA or H_2_O (Mock) 3 h before protein extraction. The protein extraction and western blot protocol as described above.

### Recombinant protein expression and purification *in vitro*

To construct recombinant protein expression strains, the *MBP*-*YFP-ECT9*, *MBP*-*YFP-ECT9N*, *MBP*-*YFP-ECT9C*, and *MBP-CFP-ECT1* plasmids were transformed into *E. coli* Rosetta (DE3) competent cells. The bacteria strains were cultured in terrific broth supplied with carbenicillin (50 mg•L-1) for 6 hours at 25 °C on a sharker of 250 rpm until OD_600 nm_ = 1, and then induced by 0.5 mM isopropyl β-D-1-thiogalactopyranoside (IPTG) for 18 h at 16°C. Cells were then spun down, resuspended in lysis buffer (20 mM Tris-HCl pH 7.4, 1 mM EDTA, 500 mM NaCl, 10 mM 2-Mercaptoethanol, 1 mM PMSF), and lysed with a high-pressure homogenizer (ATS Engineering, FB-110X). The cell lysate was centrifuged at 15,000 g for 30 min at 4 °C. The fusion protein in the soluble supernatant was purified with Dexrein Beads 6FF (Smart-Lifesciences, SA026010) according to the manufacturer’s instructions. MBP tag was removed by incubation with 10 µg•mL-1 Factor *Xa* protease (NEB, P8010S) overnight at 23 °C. Protein concentration was measured using Bradford-based Easy Protein Quantitative Kit (TransGen, DQ101-01) and examined by SDS-PAGE through Coomassie brilliant blue staining. Purified protein was flash-frozen in liquid nitrogen and stored in the storage buffer (20 mM Tris-HCl pH 7.4, 1 mM EDTA, 50 mM NaCl) at -80 °C until use.

### Phase separation assay and FRAP

Phase separation assay *in vitro* was performed with 28 µM purified protein (full length and truncated ECT9) in the storage buffer. Phase separation was induced by adding PEG8000 (Sigma, BCCC7539) at a final concentration of 20% (w/v) or reversed by adding NaCl at a final concentration of 2.5 M. Samples were dropped to a confocal dish and observed with Leica TCS SP8 upright microscopy equipped with 63× oil immersion objective using a 514 nm laser for excitation and 525-580 nm filter for emission. Bright field was captured by a differential interference contrast microscopy (Leica DMC4500) with HC PL FLUOTAR 40× objective. Turbidity was measured as optical density at 600 nm with a 500 µL volume using Nanodrop One Microvolume Spectrophotometer (Thermo Fisher Scientific).

FRAP was performed on a two-photon laser scanning fluorescence confocal microscopy (Leica, TCS SP8). For the *in vivo* experiments, FRAP of mYFP-ECT9 puncta formed in *N. benthamiana* was performed using a 40× objective lens. Bleaching was done using a 514 nm laser pulse (2 iterations, 60% intensity), and recovery was recorded for 30 pictures (a total of 39 s 18 ms) after bleaching. Ten regions of interest (ROIs) were defined for *in vivo* experiments, and eight were used for calculation. For *in vitro* FRAP analysis, technology was the same as *in vivo* experiments except for 20 pictures (a total of 26 s 21 ms) were recorded after bleaching, and four ROIs were calculated for FRAP of mYFP-ECT9N and mYFP-ECT9C floccules. The recovery curves were analyzed using GraphPad Prism 8.

### RNA extraction and real-time qPCR

For gene expression analysis in SA treatment, tobacco leaves infiltrated with *35S:ECT9-mYFP agrobacterium* was sampled for total RNA extraction with and without 2 mM SA treatment. The mYFP expression level is equally regarded as *ECT9*, and the Basta resistance gene *bar* was used as an internal control. The *A. thaliana* mutants were sampled for knock-down genes analysis. All tissues were immediately placed in liquid nitrogen and stored at -80 °C. Total RNA was extracted using the RNA isolation Total RNA Extraction Reagent Kit (Vazyme, R401-01), and 1 µg of total RNA was subjected to reverse transcription using the HiScript^®^ III 1st Strand cDNA Synthesis Kit (Vazyme, R312-01). Quantitative RT-PCR was performed using a ChamQ Universal SYBR qPCR Master Mix (Vazyme, Q711-02) on a CFX Connect Real-Time PCR Detection System (BIO-RAD). *UBIQUITIN5* (*UBQ5*) (AT3G62250) was used as an internal control for sample normalization in the gene expression analysis (2^-ΔΔCt^ method) (Schmittgen and Livak, 2008). The primers used are listed in **Supplemental Table 1**.

### Bacterial infections and ion leakage analysis

The *Psm* ES4326 and *Psm* ES4326 (AvrRpt2) strains were streak-cultured overnight at 28 °C in King-Bertani medium and screened with streptomycin (Str, 100 mg•L-1) and Str + tetracycline (Tet, 10 mg•L-1), respectively. Bacterial colonies were collected and resuspended in 10 mM MgCl_2_ and adjusted to an OD_600 nm_ of 0.001 for ETI and PTI assay. The 21-25-day-old *A. thaliana* leaves were infiltrated with the bacteria by a needleless syringe and dried with KiWi paper and inoculated plants were kept under a short-day greenhouse for 72 h and then sampled for bacterial growth. Three leaves were punched using a hole puncher as one biological replicate and 8 biological replicates for each genotype. For PTI, plants were infiltrated with 1 µM elf18 or H_2_O one day earlier than a bacterial infection. The number of colonies (CFU per drop) was calculated, and bacterial growth was represented as CFU•cm-2 of leaf tissue. For ion leakage measurement, leaves were infiltrated with *Psm* ES4326 (AvrRpt2) of OD_600 nm_ of 0.02 before 10 am and waited for absorption for 2 h. Subsequently, six leaf discs per replicate were transferred to a 50 mL sterile conical tube containing 6 mL distilled water followed by washing with 50 mL H_2_O up and down and 4 biological replicates for each genotype. The conductivity was determined at appointed times using an Orion Star A222 Conductivity Portable Meter (Thermo Fisher Scientific, K13720) and analyzed using GraphPad Prism 8.

### Callose quantification

Callose deposition was quantified by aniline blue staining and microscopy analysis described previously (Luna et al., 2011). Briefly, 4-week-old Arabidopsis leaves were infiltrated with 1 µM elf18 and incubated for 24 h. Leaf discs were then collected and soaked in 95% ethanol until all tissues were transparent. After washing with distilled water for 30 minutes, leaf discs were incubated overnight in 0.07 M phosphate buffer containing 0.01% aniline blue (Sangon Biotech, 28632-66-5) by gently shaking before microscopic analysis. Observations were performed using Leica TCS SP8 two-photon laser scanning fluorescence confocal microscopy equipped with a UV filter (excitation/emission: 405 nm/420-480 nm).

### RNA-seq analysis

Two biological replicates of Col-0 and *ect1/9* mutant leaves were collected 24 h after pressure infiltration with *Psm* ES4326 (AvrRpt2) (OD_600 nm_ = 0.001). For each sample, equal amounts of RNA from two biological replicates were pooled for RNA-seq library construction. Sequencing was performed on an Illumina NovaSeq PE150 platform with 150-bp single-end reads (Novogene). All the downstream analyses were based on clean data with high quality. Reads were mapped to the Arabidopsis TAIR10 genome. Differential expression analysis (DEGs) of Mock and ETI treatment was performed using the DESeq2 package (1.34.0) and graphically represented in a volcano plot by GraphPad Prism 8. The DEGs were identified with the criteria set as P-adjust < 0.05 and fold change > 2. Two valid biological replicates were carried out for the transcriptomic analysis. GO analysis was performed and plotted on ShinyGO 0.76.3 (“http://bioinformatics.sdstate.edu/go/“). Venn diagrams were generated using the web tool Draw Venn Diagram (“http://bioinformatics.psb.ugent.be/webtools/Venn/“). A description of the DEGs of each sample is listed in **Supplemental Table 3**.

## Data availability statement

The original contributions presented in the study are publicly available. This data can be found under accession no. PRJNA917434 on the National Center for Biotechnology Information (NCBI) Sequence Read Archive (SRA).

## Author contributions

HW, GZ and GX designed the experiments. HW and GZ performed the experiments. RN analyzed the RNA-seq data. YZ and ZT helped with the data analysis. HW, GZ and GX wrote the manuscript with input from all authors. All authors contributed to the article and approved the submitted version.
