# Supplementary material for: ECT9 condensates with ECT1 and regulates plant immunity
Source: Front Plant Sci. 2023 Apr 11;14:1140840. doi: 10.3389/fpls.2023.1140840 (PMC10126281; doi:10.3389/fpls.2023.1140840)
Supplement: Supplementary file 1 [file DataSheet_1.zip › Suppplementary data/Supplementary Material.docx]

Supplementary Material

**ECT9 condensates with ECT1 and regulates plant immunity**

**Hui Wang^1^, Ruixia Niu^1^, Yulu Zhou^1^, Zhijuan Tang^1^, Guoyong Xu****^1,2^*, Guilong Zhou^1^***

***Correspondence:**

Dr. Guoyong Xu: [guoyong.xu@whu.edu.cn](mailto:guoyong.xu@whu.edu.cn)

Dr. Guilong Zhou: [guilong.zhou@whu.edu.cn](mailto:guilong.zhou@whu.edu.cn)

1. **Supplementary Figures and Tables**

**1.1 Supplementary Figures**

**Supplementary Figure 1.** Phylogenetic analysis and subcellular localization of Arabidopsis YTH domain-containing proteins. (A) Phylogenetic analysis of 160 YTH proteins from 11 species, including *Arabidopsis thaliana* (At, Taxonomy ID: 3702), *Oryza sativa* (Os, 4530), *Zea mays* (Zm, 4577), *Glycine max* (Gm, 3847), *Triticum aestivum* (Ta, 4565), *Hordeum vulgare* (Hv, 4513), *Sorghum bicolor* (Sb, 4558), *Gossypium raimondii* (Gr, 29730), *Brachypodium distachyon* (Bd, 15368), *Homo sapiens* (HUMAN, 9606), Yeast (*Saccharomyces cerevisiae* (Pho92), 4932; *Schizosaccharomyces pombe* (MMI1), 4896). The YTH domain sequence of each protein was used for multiple sequence alignment and phylogenetic tree construction. The two clades, YTHDF subfamily and YTHDC subfamily, are indicated. (B) Phylogenetic analysis and domain/genome architectures of 13 Arabidopsis YTH proteins. The two clades, YTHDF subfamily and YTHDC subfamily, are indicated. Data of domain sequences and DNA sequences are organized from UniProt and TAIR websites using genes ID, respectively. The disordered score was predicted from the PONDR (<http://www.pondr.com/>) website with a VSL2 predictor using protein sequences. Subcellular localization, SA responsiveness and LLPS were observed in *N. benthamiana* in this study. (C) Subcellular localization of 13 Arabidopsis YTH proteins in *N. benthamiana* leaves expressing 35S promotor-driven fusion vectors. Scale bar, 10 µm. Data are representative of three independent experiments.

**Supplementary Figure 2.** Co-localization of ECT9 condensates with body markers (A) and in Arabidopsis protoplasts (B). (A) Co-localization of ECT9 with three known body markers. The ECT9-CFP fusion protein was co-expressed with SG marker UBP1b-mYFP, cytoplasmic body marker G3BP1-mYFP and nuclear body marker FCA-mYFP in *N. benthamiana* leaves. Scale bar, 10 µm. The right panels show relative fluorescence intensity plots of the CFP (red) or mYFP (green) along the line from a to b depicted in the corresponding merged images. Solid white line rectangles indicate the area considered to calculate the co-localization score (*P*, Pearson’s R-value) depicted at the bottom-left of merged images. **(**B**)** Confocal laser scanning microscopy (CLSM) images of mYFP fluorescence were photographed for *35S:ECT9-mYFP* transient expression into wild type Arabidopsis mesophyll protoplasts under Mock (H_2_O) or 2 mM SA treatment. Scale bar, 10 µm. Data are representative of three independent experiments.

**Supplementary Figure 3.** Truncated ECT9 proteins lose the ability to form liquid-like droplets. (A) Schematic of truncated protein fusions used for in vitro phase separation assay. (B) Phase separation assay of about 28 µM mYFP-ECT9N and mYFP-ECT9C aggregates by confocal laser scanning microscopy photograph. Scale bar, 25 µm. (C) FRAP of mYFP-ECT9N and mYFP-ECT9C aggregates in vitro. Time 0 indicates the time of the photobleaching pulse. Scale bar, 5 µm. Plot showing the time course of the recovery after photobleaching mYFP-ECT9N and mYFP-ECT9C aggregates. Mean ± SD (n = 4). (D) Subcellular localization of truncated proteins (ECT9N and ECT9C) in *N. benthamiana* leaves. Scale bar, 10 µm. Data are representative of three independent experiments.

**Supplementary Figure 4.** Identification and expression analysis of transgenic mutans. (A) Construction of CRISPR/Cas9-based *ect1* and *ect9* knockout transgenic lines. Two gRNA sequences (gRNA1 and gRNA2) that specifically target *ECT*1 and *ECT9* are used, thus generating a deletion mutation of 922 bp and 1791 bp in *ECT*1 and *ECT9* genome, respectively. Homozygous mutants were identified by PCR using gene specific primers (F and R) and further sequenced. Diagram of agarose gel electrophoresis shows the results of a PCR reaction for edit events detection. (B) RNA expression level of *ECT1* and *ECT9* in *ect1*, *ect9* and *ect1/9* mutants determined by RT-qPCR using gene specific primers. The data represents mean ± SD calculated from four technologic replicates. (C) The developmental phenotype of Col-0, *ect1*, *ect9* and *ect1/9* mutants. The leaves shown were photographed at 24 days. Scale bar, 3 cm.

**Supplementary Figure 5.** ECT1 and ECT9 are not the essential immune components in basal resistance and PTI. (A) Measurement of bacterial counts in the leaves of Col-0, *ect1*, *ect9*, *ect1/9* and *npr1* plants after infiltration with *Psm* ES4326 at OD_600_ _nm_ = 0.0001 at 3 dpi (mean ± SD; n = 5). Data were analyzed using one-way ANOVA with Dunnett’s test. dpi, days post-inoculation. (B) Growth of *Psm* ES4326 on the indicated genotypes. *Psm* ES4326 was infiltrated into Arabidopsis leaves at OD_600_ _nm_ = 0.001 after 1 day of treatment with H_2_O or 1 µM elf18 and populations were determined at 3 dpi (mean ± SD; n = 7). Data were analyzed using two-way ANOVA with Bonferroni’s test. dpi, days post-inoculation. (C) Morphologic differences between callose depositions of Col-0, *ect1*, *ect9*, *ect1/9* and *efr* plants. Callose was stained with aniline blue 24 h after treatment with 1 µM elf18. Scale bar, 100 µm. Data are representative of three independent experiments. (D) Measurement of bacterial counts in the leaves of Col-0, *rps2*, *ect1 ect9* and complementary lines after infiltration with *Psm* ES4326 (AvrRpt2) at OD_600_ _nm_ = 0.001 at 3 dpi (mean ± SD; n = 7). Data were analyzed using one-way ANOVA with Dunnett’s test. dpi, days post-inoculation.

**Supplementary Figure 6.** Assessment of reproducibility for RNA-seq datasets. Pearson correlation coefficient (*r*) of read counts for protein-coding genes with a FPKM > 1 between the two biological replicates of each sample was conducted in R studio. The high correlation coefficients observed across biological replicates (*r* = 0.98) indicate high biological reproducibility (that is, low biological variation).

**1.2 Supplementary Tables**

**Supplementary Table 1. Plasmids and Primers used in this study.**

**Supplementary Table 2. YTH domain sequences of 11 species.**

**Supplementary Table 3. Analysis of RNA-seq datasets.**

**Supplementary Table 4. SLCA of ECT9 and other YTHs.**
