# Supplementary figures and images for: ECT9 condensates with ECT1 and regulates plant immunity
Source: Front Plant Sci. 2023 Apr 11;14:1140840. doi: 10.3389/fpls.2023.1140840 (PMC10126281; doi:10.3389/fpls.2023.1140840)

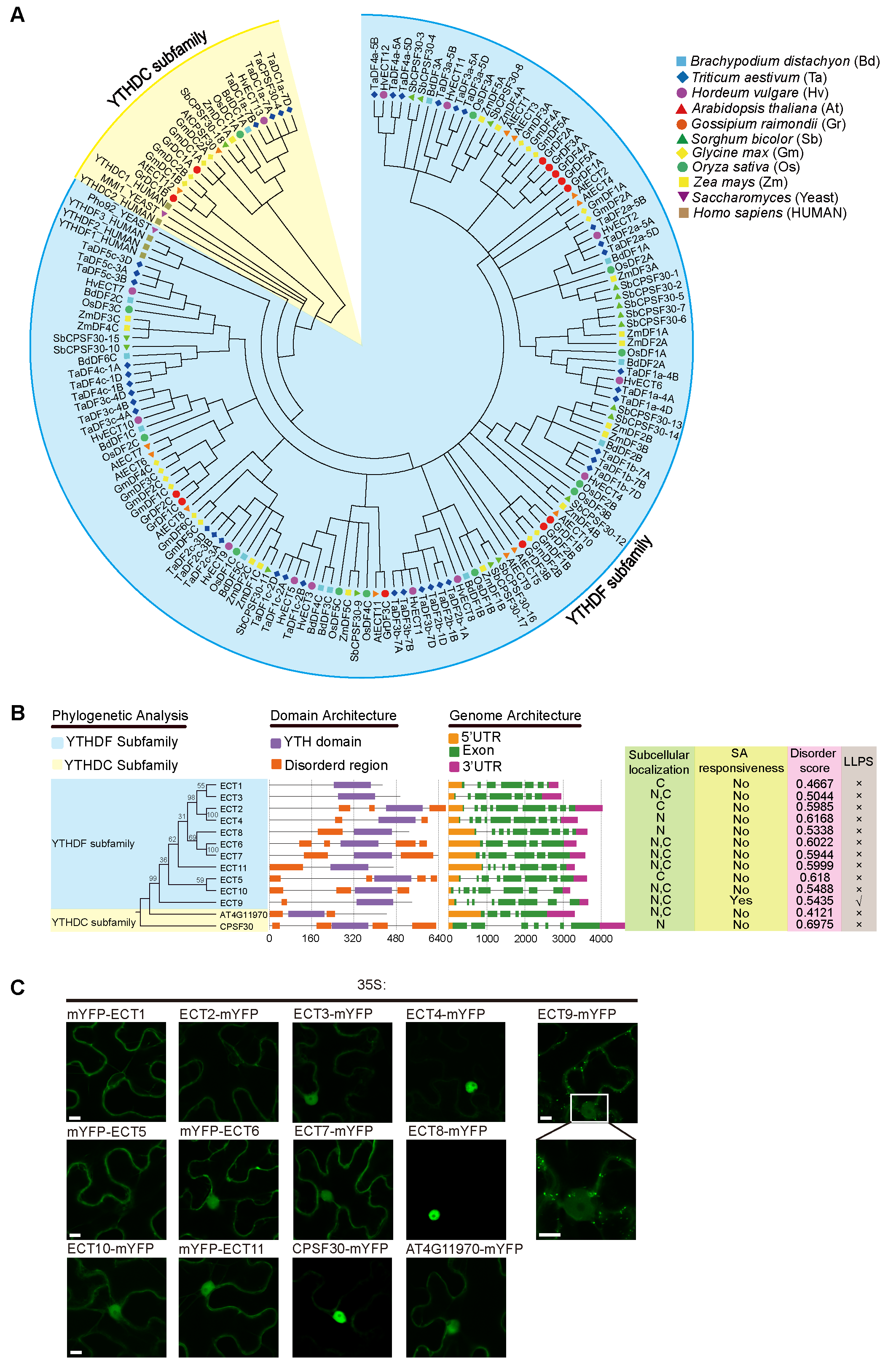

Supplement: Supplementary file 2 [file Image_1.tif]

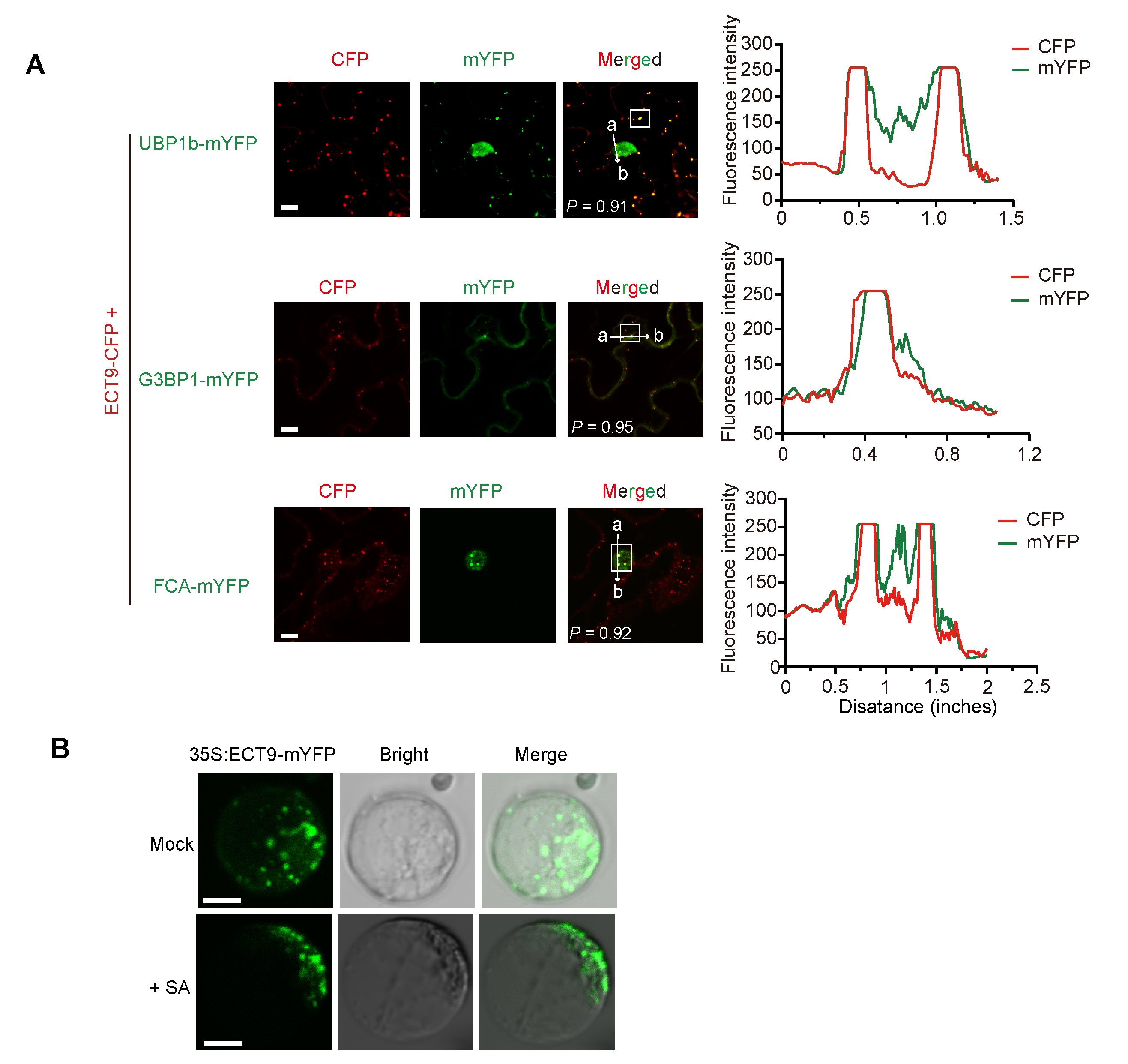

Supplement: Supplementary file 3 [file Image_2.tif]

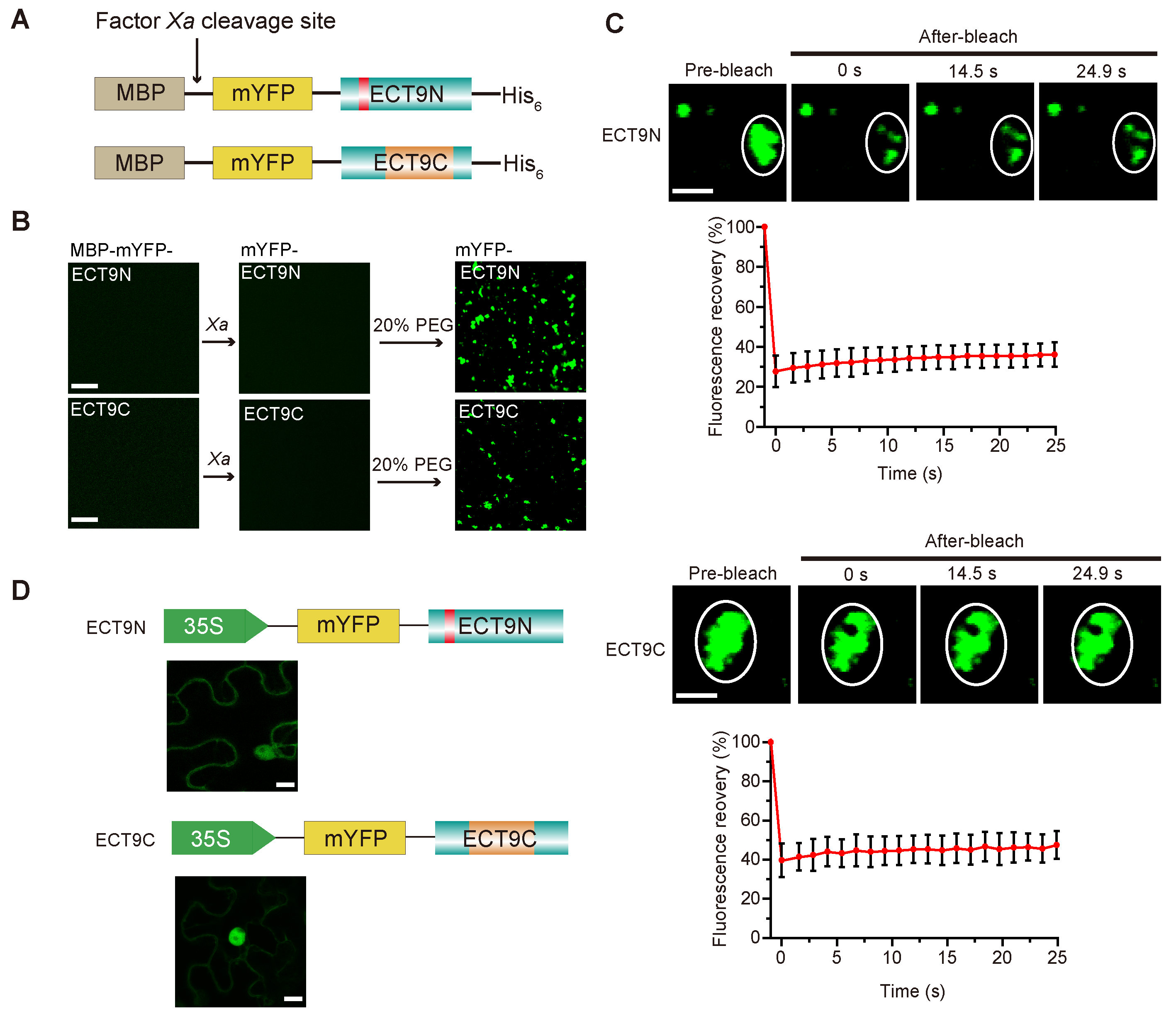

Supplement: Supplementary file 4 [file Image_3.tif]

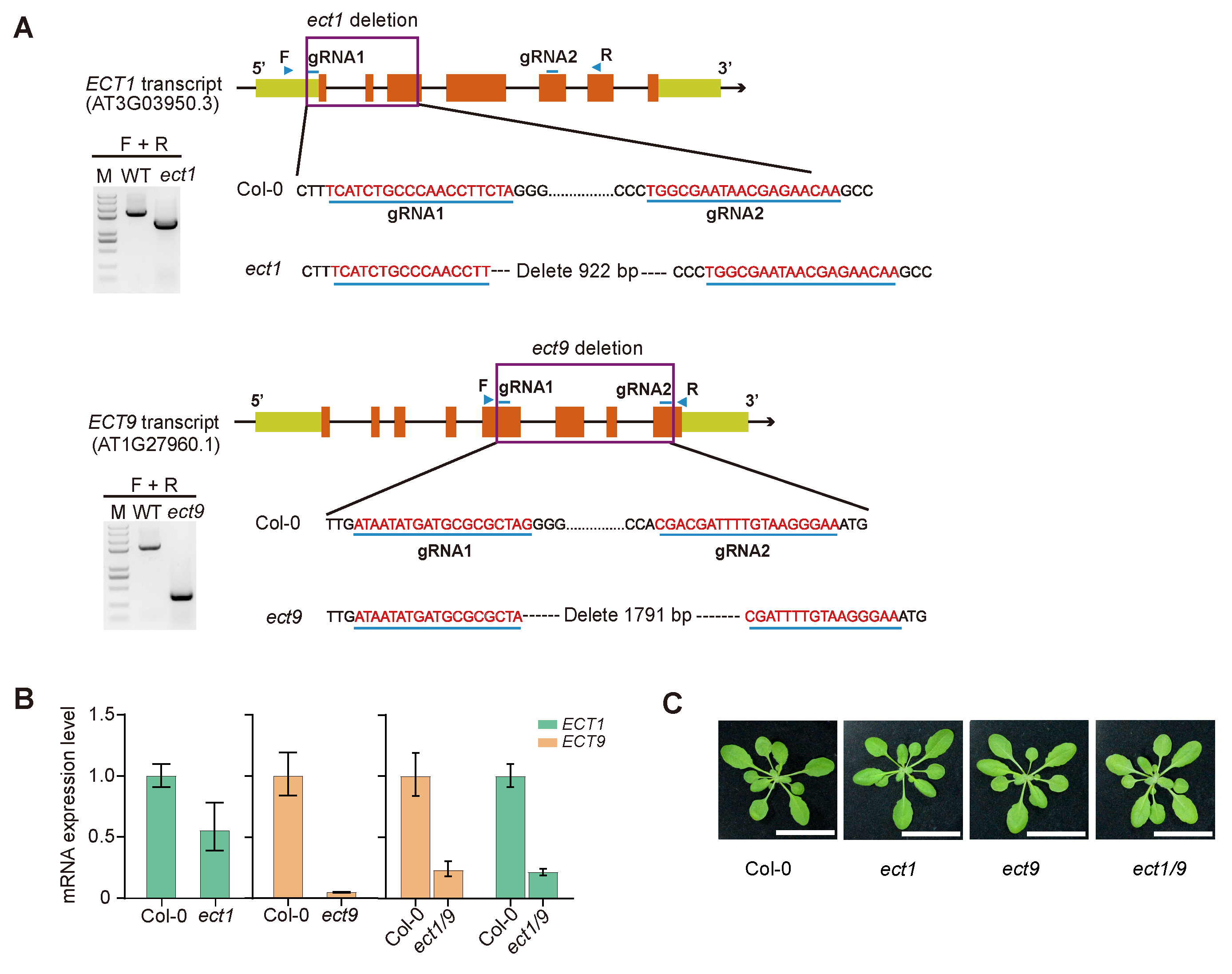

Supplement: Supplementary file 5 [file Image_4.tif]

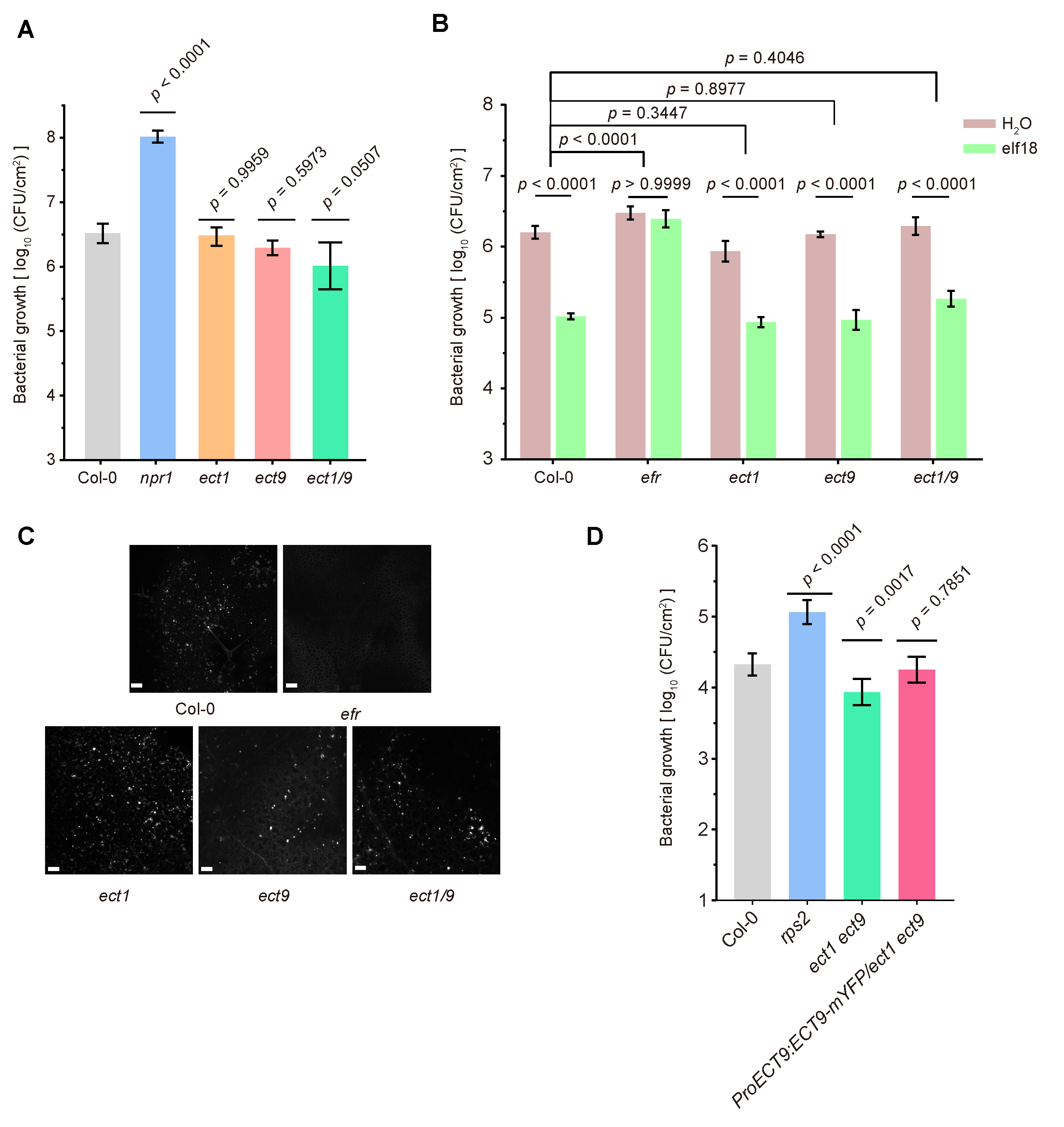

Supplement: Supplementary file 6 [file Image_5.tif]

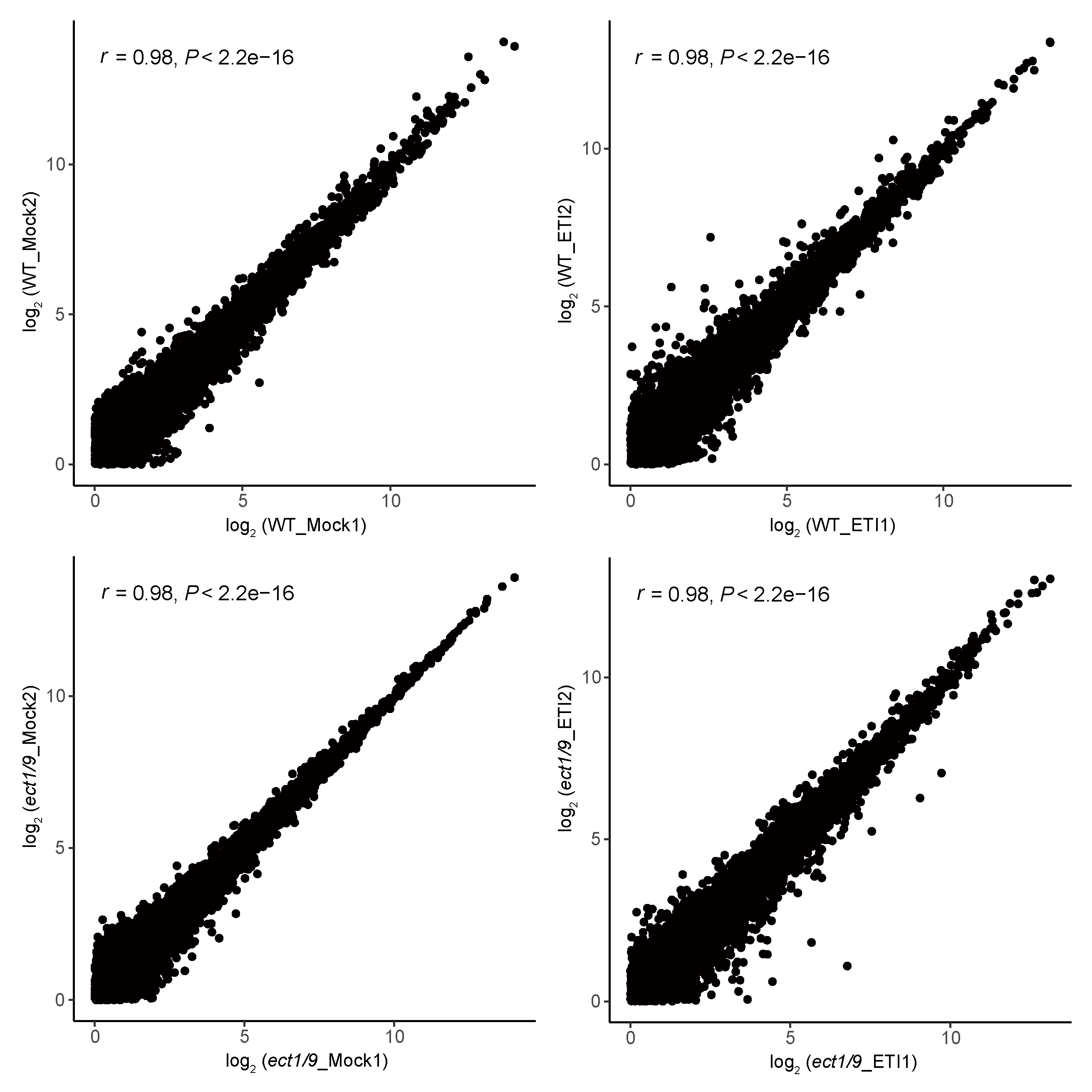

Supplement: Supplementary file 7 [file Image_6.tif]
